# Validation of the Patient-Doctor-Relationship Questionnaire (PDRQ-9) in a Representative Cross-Sectional German Population Survey

**DOI:** 10.1371/journal.pone.0091964

**Published:** 2014-03-17

**Authors:** Markus Zenger, Rainer Schaefert, Christina van der Feltz-Cornelis, Elmar Brähler, Winfried Häuser

**Affiliations:** 1 Department of Medical Psychology and Medical Sociology, University of Leipzig, Leipzig, Germany; 2 Department of General Internal Medicine and Psychosomatics, University of Heidelberg, Heidelberg, Germany; 3 Tranzo Department, Faculty of Social Sciences of the University of Tilburg, Tilburg; Clinical Center for Body, Mind and Health, Tilburg, The Netherlands; 4 Trimbos Instituut, Utrecht, The Netherlands; 5 Department of Psychosomatic Medicine and Psychotherapy, University Medical Center of the Johannes Gutenberg-University, Mainz, Germany; 6 Department of Internal Medicine I, Klinikum Saarbrücken, Saarbrücken, Germany; 7 Department of Psychosomatic Medicine, Technische Universität München, München, Germany; University of Texas Health Science Center at Houston, United States of America

## Abstract

The patient-doctor relationship (PDR) as perceived by the patient is an important concept in primary care and psychotherapy. The PDR Questionnaire (PDRQ-9) provides a brief measure of the therapeutic aspects of the PDR in primary care.

We assessed the internal and external validity of the German version of the PDRQ-9 in a representative cross-sectional German population survey that included 2,275 persons aged≥14 years who reported consulting with a primary care physician (PCP).

The acceptance of the German version of this questionnaire was good. Confirmatory factor analysis demonstrated that the PRDQ-9 was unidimensional. The internal reliability (Cronbach's α) of the total score was .95. The corrected item-total correlations were≥.94. The mean satisfaction index of persons with a probable depressive disorder was lower than that of persons without a probable depressive disorder, indicating good discriminative concurrent criterion validity. The correlation coefficient between satisfaction with PDR and satisfaction with pain therapy was r = .51 in 489 persons who reported chronic pain, indicating good convergent validity. Despite the limitation of low variance in the PDRQ-9 total scores, the results indicate that the German version of the PDRQ-9 is a brief questionnaire with good psychometric properties to assess German patients' perceived therapeutic alliance with PCPs in public health research.

## Introduction

The patient-doctor relationship (PDR) is an important concept in health care. A good physician-patient relationship is associated with better treatment adherence, higher patient satisfaction, and a better prognosis [1]–[4]. Several aspects of the PDR have commonalties with the helping alliance in psychotherapy, i.e., high levels of trust, helpfulness, empathic understanding, and interpersonal openness [5]. Both the patient's and the physician's perspectives must be considered to understand the PDR [6]. Substantial efforts have been made to develop instruments to assess the PDR from the patient's point of view. A systematic review found 19 instruments that assess the PDR. These instruments assessed a variety of dimensions and used diverse conceptual models for the PDR [7]. The authors stated that in the primary care setting, a research instrument is preferably concise and easy to use. They suggested the use of the Patient-Doctor Relationship Questionnaire (PRDQ-9) as a brief (9 items) questionnaire with excellent overall internal consistency [7].

The Patient-Doctor Relationship Questionnaire (PRDQ-9) was originally developed in the Netherlands as a short assessment of the relationship between the primary care physician (PCP) and the patient from the patient's perspective [8]. It adapted an existing instrument from psychotherapeutic research on therapeutic alliance, the Helping Alliance Questionnaire (HAQ) [9], for use in primary care and public health research. The HAQ contains 11 items and served as the basis for item creation and selection in the PDRQ. In adapting the instrument to the needs of primary care, some strongly psychotherapeutic aspects (e.g., gaining new insight) were omitted or rephrased, and other aspects (e.g., ‘My PCP has enough time for me’, ‘My PCP is dedicated to help me’) were added. This procedure resulted in the first, 15-item version of the PDRQ. The psychometric properties of the PDRQ were initially tested in a rather small sample of 110 general practice patients and 55 patients in an epilepsy clinic [8]. In this validation study, a principal component factor analysis with varimax rotation of the 15 items resulted in 2 factors. The first factor focused on the empathic style and availability of the doctor and accounted for 58% of the total variance explained. The second factor focused on the medical symptoms of the patients and accounted for 9% of the total variance explained. The internal consistency of the first factor was high and that of the second was moderate. With the aim of clearly assessing the patient-doctor relationship with a focus on the empathic style and availability of the doctor, the second factor was eliminated. This resulted in the final, unidimensional 9-item version of the PRDQ-9, with all 9 items loading onto 1 common factor [8]. A mean satisfaction index of all 9 items can be calculated [8]. Validation studies of a Spanish version comprised 188 patients of 6 internal medicine physicians of a university hospital [10] and 405 patients of 6 primary health care centers [11]. A validation study of a Turkish version was performed with 405 patients of a family medicine outpatient center [12].

To date, the psychometric properties of the PDRQ-9 have not been tested in a larger sample of the general population within the setting of public health research. Furthermore, a version for German-speaking patients has not yet been validated. Therefore, the aim of the present study was to test the internal and external validity of the German version of the PDRQ-9 in a representative general population sample.

## Methods

### 1. Ethics statement

All participants were informed of the study procedures, data collection and anonymization of all personal data. Furthermore, a detailed data privacy statement was delivered by the study assistant. The present study posed a low risk to the participants, as procedures such as medical treatments, invasive diagnostics or procedures causing psychological, spiritual or social harm were not included in the present study. Therefore, according to the German law, all participants provided verbal informed consent, which was noted by the trained interviewer before starting with the survey. The additional informed consent of a parent was not required for participants aged 14 or older. The study and procedure, including the consent procedure, were approved by the institutional ethics review board of the University of Leipzig (Az 092-12-05032012). Furthermore, the study adhered to the guidelines of the ICC/ESOMAR International Code of Marketing and Social Research Practice.

### 2. Linguistic adaptation

The PDRQ-9 was first developed in Dutch. As performed in the Spanish [11] and Turkish [12] validation studies, the PDRQ-9 was adapted to German by translating it from its primarily published [8] and used English version. The adaptation to German was performed according to the state-of-the-art procedure of forward-backward translation [13] by 2 medical doctors and 1 English-German bilingual translator. Two forward translations into German were independently completed by 2 medical doctors, both of whom are native speakers of the German language and are fluent in English. The 2 German versions were compared, and an updated German forward version was compiled. This version was translated back into English by a professional translator (a native speaker of English who is fluent in German) with experience in medical translation. This translator had not been involved in the forward translation. The primarily published version and the back-translated version – both in English – were compared by the 2 medical doctors and the expert translator. Thus, an optimized German version was generated. Additionally, this optimized German version was compared with the original Dutch instrument by the German-speaking first author of the PDRQ-9 (van der Feltz-Cornelis), whose native language is Dutch. In a final reconciliation process, the final German version (PDRQ-9 German, see Appendix S1) was generated and approved by all parties. All comparisons between the different versions were conducted item-by-item on 2 dimensions: similarity of language (literal translation) and comparability of interpretation (cultural adaptation). Discrepancies and discussions mainly regarded 2 items. For item 6, the consensus was to translate “nature” as “Wesen” (rather than “Natur”). For item 9, the consensus was to translate “easy accessible” as “leicht zu erreichen” to emphasize organizational rather than emotional accessibility. The measure was not pilot tested before being employed in the full study, as such testing is not a typical step in forward-backward translation.

### 3. Design and participants

The current study was part of the 2013 annual representative general population survey that was conducted by the University of Leipzig. This survey assessed political and religious attitudes as well as health topics.

A representative sample of the German population was selected with the assistance of a demographic consulting company (USUMA, Berlin, Germany). The random selection was based on multistage sampling. First, 258 sample point regions, covering rural and urban areas from all regions in Germany, were randomly drawn from the most recent political election register. The second stage was a random selection of households using the random route procedure (based on a starting address). The third stage was a random selection of household respondents using the Kish selection grid. The aim of the sampling procedure was to obtain a sample that was representative of the German population in terms of age, gender, and education. The inclusion criteria for the study were age≥14 years and the ability to read and understand the German language.

All subjects were visited by a trained study assistant and informed about the investigation. The subjects were provided with self-rating questionnaires. The survey included several questionnaires on somatic and psychological features (health survey) as well as questionnaires on eating behavior, political attitudes and media use. The survey also asked the participants whether they had a PCP. In the case of a positive response to this question, the person was asked to complete the PDRQ-9. The assistant was available while the participants answered all of the questionnaires and offered help if persons did not understand the meaning of any question. Regarding the questionnaires used in the current study, the trained assistants did not report any systematic misunderstanding of the items.

### 4. Validation methods and hypotheses

The methods used to validate the PDRQ-9 German were as follows:

a) Acceptance was assessed according to the proportion of missing or invalid items.

b) Data quality was assessed using the mean, median and extent of ceiling and floor effects. Floor and ceiling effects between 1% and 15% were defined as optimal [14].

c) Reliability was assessed as internal consistency (Cronbach's α), which measures the overall correlation between items within a scale. A level of .7 and higher is considered desirable [15].

d) Factorial structure was tested using confirmatory factor analysis (CFA).

e) Convergent validity was determined by comparing the mean satisfaction index of the PDRQ-9 with the treatment satisfaction ratings of persons in the general population with chronic pain [16]. We expected a positive correlation between these 2 satisfaction indices. The convergent validity is considered fulfilled if the scale scores for related concepts show acceptable correlation (Spearman rank correlation coefficient>.4) [15].

f) Discriminative concurrent criterion validity was tested by comparing the PDRQ-9 total score of persons in the general population with a probable depressive disorder (PHQ-2≥3) to persons without a probable depressive disorder. We predicted that participants with a probable depressive disorder would report a lower mean satisfaction index than persons without a probable depressive disorder [17]. This hypothesis was based on the cognitive theory of depression. The cognitive triad of depression is characterized by dysfunctional negative views of oneself, one's life experience (and the world in general), and one's future [18]. We assumed that this negative view would also apply to the PDR.

g) Potential associations with socioeconomic variables (age, gender, education, and household income) were tested using multiple linear regression analysis.

### 5. Validation instruments

#### 5.1 Demographic questionnaire

Age, gender, partnership status, educational level, employment status, and net family income per month were assessed via a standardized questionnaire that was previously used in German health surveys [19].

#### 5.2 Chronic pain questionnaire

Individuals with chronic non-cancer pain were identified by screening questions based on the International Association of the Study of Pain (IASP) definition of chronic pain [16], as follows: “Did you have constant or frequently recurring pain during the last 3 months?” In the case of self-reported current treatment of chronic pain, participants were asked to report their satisfaction with pain treatment (1 = very unsatisfied, 2 = unsatisfied, 3 = satisfied, 4 = very satisfied).

#### 5.3 Depression screening questionnaire

The 2-item Patient Health Questionnaire-2 (PHQ-2) scores 2 DSM-IV criteria of major depression on a scale from “0” (not at all) to “3” (nearly every day) [20]. A score≥3 on this depression scale represents a reasonable cut-off for identifying potential cases of major depression or other depressive disorders. A score≥3 has a sensitivity of 82.9% and a specificity of 90% for the diagnosis of major depression and a sensitivity of 62.3% and a specificity of 94% for the diagnosis of any depressive disorder. We used the validated German version of the PHQ-2 [21].

### 6. Statistical analyses

We prespecified that up to 2 missing items on an individual's PRDQ-9 would be replaced by the rounded mean of the answered items. If more than 2 items of the scale remained unanswered, the respective person was excluded from further analyses. In addition, descriptive statistics were performed to determine whether a specific item on the German version had many missing values because this might indicate insufficient understanding of the translation of that item.

Because Cronbach's α represents a lower bound estimate of reliability, a composite reliability (CR) score and the average variance extracted (AVE), according to Fornell and Larcker [22], were also calculated.

The factorial structure was tested using CFA, which was computed with the statistical program AMOS 20 (IBM SPSS Inc., Chicago, IL, 2011). The model was tested using covariance matrices and estimated with the maximum likelihood approach. CFA was calculated for the one-factor model. The following model fit indices were used: the minimum discrepancy divided by its degrees of freedom (CMIN/DF); the goodness-of-fit index (GFI); the normed fit index (NFI); the comparative fit index (CFI); the Tucker-Lewis Index (TLI); the standardized root mean square residual (SRMR); and the root mean square error of approximation (RMSEA). For a good model fit, the CMIN/DF ratio should be as small as possible [23], [24] and the CFI should range between .97 and 1 [24]. Furthermore, GFI, NFI and TLI values that are near .95 or higher are indicative of a good model fit [24], [25]. An SRMR value that is smaller than .05 [23], [24] and an RMSEA value that is .08 or smaller indicate an adequate fit [24]. Additional analyses were conducted to test the invariance of the model across gender and different age groups using multi-group CFA. Age groups were defined based on age decades and substantial subsample sizes to conduct the analyses. Therefore, participants in the age range between 14 and 30 years were categorized into the same age group. Measurement invariance was tested in 4 steps using the configural model (no constraints), followed by a metric invariant model (with item loadings constrained to be equal across groups), a scalar invariant model (with item loadings and item intercepts simultaneously constrained to be equal across groups), and a model of strict factorial invariance (with error variances constrained to be equal across groups in addition to the conditions mentioned above) [26]. Following the hierarchy of these nested and increasingly restrictive models, they were compared to each other based on the ΔCFI and ΔRMSEA, as the χ^2^ statistic has often been criticized for its sensitivity to the sample size. Values that are smaller than .01 indicate the invariance of the models [27]. These invariance tests are mandatory in a statistical manner to allow further tests of mean differences between the defined sub-groups [26].

The remaining statistical analyses were conducted using IBM SPSS version 20. Group comparisons were performed by ANOVAs and ANCOVAs. The ANCOVA effect sizes were expressed as partial η^2^, which was interpreted as a small effect size when≥.01, a medium effect size when≥.06 and a large effect size when≥.13. Partial η^2^ describes the proportion of total variation that is attributable to the factor, excluding other factors from the nonerror variation [28]. The data are available upon request.

## Results

### 1. Sample recruitment and response rate

**Figure 1:**
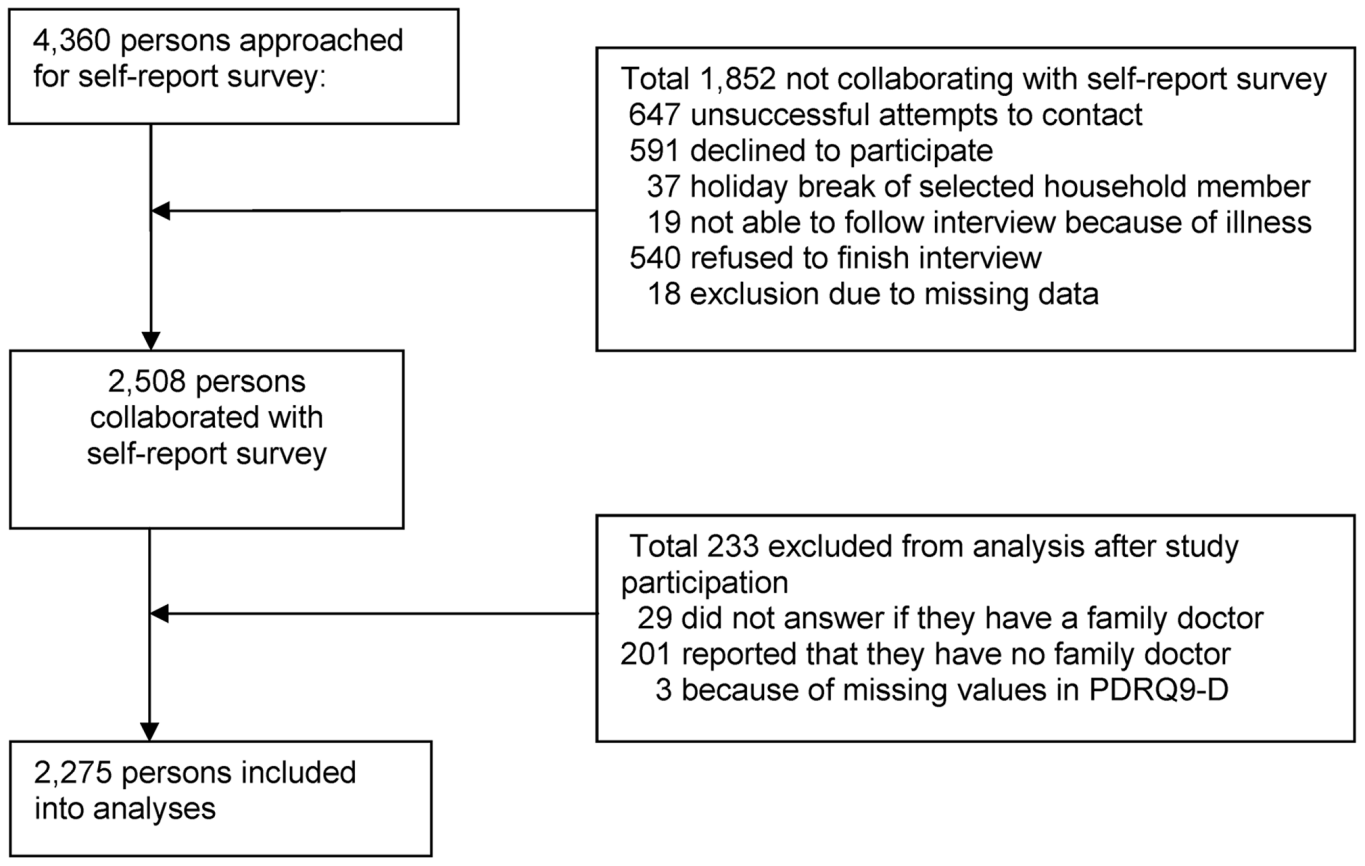
Study flow diagram.

Data were collected between May and June 2013. A first attempt was made at 4,360 addresses, and 2,508 (57.5%) persons participated in this self-report survey. The inclusion and exclusion of participants for the final analyses are shown in the flow chart (Figure 1). Overall, 2,275 (52.2%) persons were included in the final analyses.

### 2. Sample characteristics

The demographic characteristics of the study population are presented in Table 1. The study sample displayed age groups, sex ratio and educational levels that were comparable to those of the general German population, as assessed by the German population census in 2011 [29].

**Table 1:** Demographic characteristics of the study population.

	Total N = 2,275	Men N = 1,031	Women N = 1,244
*Age M (SD)*	50.74 (18.22)	50.58 (18.07)	50.87 (18.35)
*Age range*	14–92	14–92	14–92
*Age groups*	**N (%)**	**N (%)**	**N (%)**
14–30 years	396 (17.4)	176 (17.1)	220 (17.7)
31–40 years	286 (12.6)	133 (12.9)	153 (12.3)
41–50 years	414 (18.2)	181 (17.6)	233 (18.7)
51–60 years	412 (18.1)	201 (19.5)	211 (17.0)
61–70 years	400 (17.6)	183 (17.7)	217 (17.4)
≥71 years	367 (16.1)	157 (15.2)	210 (16.9)
*Living in partnership*			
Yes	1,211 (53.2)	598 (58.0)	613 (49.3)
No	1,064 (46.8)	433 (42.0)	631 (50.7)
*Education*			
≤8 years	892 (39.2)	499 (38.7)	493 (39.6)
9–10 years	927 (40.7)	396 (38.4)	531 (42.7)
10–12 years	385 (16.9)	194 (18.8)	191 (15.3)
School student	65 (2.9)	38 (3.7)	27 (2.2)
Missing	6 (0.3)	4 (0.4)	2 (0.2)
*Employment status*			
Education/training	152 (6.7)	76 (7.4)	76 (6.1)
Working	1,112 (48.9)	557 (54.0)	557 (44.8)
Unemployed/working <15 h per week	168 (7.4)	71 (6.9)	97 (7.8)
House wife/man	111 (4.9)	5 (0.5)	106 (8.5)
Retired	730 (32.1)	322 (31.2)	408 (32.8)
*Household income in €*			
<1,500	747 (32.8)	280 (27.2)	467 (37.5)
1,500-<2,000	394 (17.3)	173 (16.8)	221 (17.8)
2,000-<2,500	377 (16.6)	185 (17.9)	192 (15.4)
≥2,500	688 (30.2)	363 (35.2)	325 (26.1)
Missing	69 (3.0)	30 (2.9)	39 (3.1)

### 3. Validity

#### 3.1 Acceptance

The acceptance was high. Only 23 (1.0%) single items were not answered, none of the participants had more than 1 missing item, and there were no items that were predominantly missing.

#### 3.2 Data quality

The means and standard deviations of all items are shown in Table 2. Additionally, supplemental materials on the item score frequency (Table S1) and frequency distribution of the PDRQ-9 total scores (Table S2) are provided.

**Table 2:** Item characteristics of the PDRQ-9 German (N = 2,275).

Item	Missing N (%)	Mean	SD	Skewness	Corrected item-total correlation
**1 My PCP helps me**	0 (0%)	4.17	0.80	−0.80	.94
**2 My PCP has enough time for me**	0 (0%)	3.84	0.95	−0.45	.95
**3 I trust my PCP**	4 (0.2%)	4.21	0.79	−0.85	.94
**4 My PCP understands me**	3 (0.1%)	4.11	0.84	−0.87	.94
**5 My PCP is dedicated to helping me**	2 (0.1%)	4.26	0.76	−0.86	.94
**6 My PCP and I agree about the nature of my medical symptoms**	13 (0.6%)	4.06	0.86	−0.67	.94
**7 I can talk to my PCP**	0 (0%)	4.14	0.87	−0.82	.94
**8 I feel content with my PCP**'**s treatment**	1 (0.1%)	4.15	0.85	−0.97	.94
**9 I find my PCP easily accessible**	0 (0%)	4.17	0.84	−0.86	.94
**Satisfaction index**	23 (1.0%)	4.12	0.70	−0.77	

Note: PCP  =  primary care physician; SD  =  standard deviation.

The mean satisfaction index was 4.12 (SD = .70) (on a scale of 1 (the worst) to 5 (the best satisfaction possible)), with a median of 4.78 (interquartile range 4.00–5.00). Four of every 10 subjects expressed the maximum possible satisfaction (“ceiling effect”). This result is underlined by the skewness of the items (Table 2). Negative values showed a clear left skewed distribution, indicating that most of the values were concentrated on the right of the mean.

#### 3.3 Internal reliability

The corrected item-total correlation coefficients indicated that all items accounted for a substantial amount of the variance of the total scale and did not differ from each other. Furthermore, the internal consistency was high (Cronbach's α = .95). In total, the explained variance was 73.4% and the CR was .96, indicating good internal consistency of the PDRQ-9 German.

#### 3.4 Factorial structure of the PDRQ-9 German

All items of the PDRQ-9 German were positively correlated, and the correlation coefficients were of a substantial amount (Table 3).

**Table 3:** Standardized factor loadings and item correlation coefficients of the PDRQ-9 German.

	Item 1	Item 2	Item 3	Item 4	Item 5	Item 6	Item 7	Item 8	Item 9
PDRQ-9	.776	.716	.842	.840	.790	.774	.833	.878	.863
Item 2	.561								
Item 3	.703	.623							
Item 4	.654	.602	.732						
Item 5	.639	.539	.673	.681					
Item 6	.601	.564	.635	.649	.638				
Item 7	.599	.610	.680	.689	.627	.658			
Item 8	.691	.606	.734	.728	.682	.673	.741		
Item 9	.635	.623	.698	.710	.681	.657	.769	.777	

The hypothesized unidimensional structure of the PDRQ-9 fit the data very well (χ^2^ (df) = 345.860 (27); CMIN/DF = 12.810; GFI = .965; NFI = .979; CFI = .980; TLI = .974; SRMR = .019; RMSEA = .072).

Only the CMIN/DF indicated a relevant deviation between the data and the model, as a value close to 3 or smaller represents appropriate models. This coefficient is sensitive to the sample size. Thus, in line with Joereskog and Soerbom (1993), we focused on the model fit indices described above (GFI, NFI, CFI, TLI, SRMR, RMSEA), which are generally independent of the sample size.

The standardized regression coefficients of the latent variable “satisfaction with the patient-doctor relationship” varied between .72 and .88 (Table 3), indicating substantial relationships between the latent variable and each of the 9 items of the PDRQ-9.

Furthermore, the model was tested for invariance across gender and age. As shown in Table 4, the multi-group analyses revealed the invariance across gender and age, as the differences in CFI and RMSEA between the hierarchical nested models were<.01. The χ^2^ test was significant for several invariance tests between different sub-groups. As mentioned above, this test is sensitive to sample size. Thus, the other fit indices were used to confirm the scalar invariance across gender and age.

**Table 4:** Test for invariance across gender and age.

	N	?^2^ (df)	Δ χ^2^	Δ df	Δ p	CMIN/DF	CFI	Δ CFI	RMSEA	Δ RMSEA
***Gender***										
Men	1,031	176.266 (27)				6.528	.979		.073	
Women	1,244	223.535 (27)				8.279	.979		.077	
***Multigroup analysis***										
Dimensional/configural		399.800 (54)				7.404	.979		.053	
Metric		404.270 (62)	4.470	8	.812	6.520	.979	.000	.049	.004
Scalar		411.797 (71)	7.527	9	.582	5.800	.979	.000	.046	.003
Strict factorial		454.029 (81)	42.231	10	<.001	5.605	.977	.002	.045	.001
***Age groups***										
14–30 years	396	99.464 (27)				3.684	.972		.082	
31–40 years	286	87.418 (27)				3.238	.974		.089	
41–50 years	414	72.266 (27)				2.677	.984		.064	
51–60 years	412	56.785 (27)				2.103	.990		.052	
61–70 years	400	146.561 (27)				5.428	.957		.105	
>70 years	367	87.739 (27)				3.250	.979		.078	
***Multigroup analysis***										
Dimensional/configural		550.256 (162)				3.397	.976		.033	
Metric		603.064 (202)	52.807	40	.085	2.985	.975	.001	.030	.003
Scalar		693.771 (247)	90.707	45	<.001	2.809	.973	.002	.028	.002
Strict factorial		827.913 (297)	134.142	50	<.001	2.788	.967	.006	.028	.000

Note: df: degrees of freedom; CMIN/DF: minimum discrepancy divided by its degrees of freedom; CFI: comparative fit index; RMSEA: root mean square error of approximation.

#### 3.5 Convergent validity

The Spearman rank correlation between the mean satisfaction index and the satisfaction with pain treatment of 489 participants who reported chronic pain and pain treatment was r = .51. This result demonstrates acceptable convergent validity for this subsample.

#### 3.6 Discriminative concurrent criterion validity

In an ANOVA that adjusted for age, the mean satisfaction index of participants with a potential depressive disorder (N = 218) was 3.66 (SD = .86), and that of participants without a potential depressive disorder (N = 2,030) was 4.12 (SD = .66) (F = 65.8, p<.001). Potential depressive disorder primarily accounted for a group difference in mean satisfaction index (F = 119, p<.0001), with a small effect size (Partial η^2^ = .05). The partial η^2^ of age was .007 (F = 7.1, p<.001). This result demonstrates acceptable discriminative concurrent criterion validity.

### 4. Associations of the PDRQ-9 German total score and socioeconomic variables

To examine the influence of socioeconomic variables on PDRQ-9 German total scores, a simultaneous multiple linear regression analysis was conducted, with age (as a continuous variable), gender, education, and household income (variables coded according to the groups presented in Table 1) as predictors. The results are presented in Table 5. The only significant predictors were age and income, with a higher satisfaction index among older patients and those with higher household income. However, the amount of explained variance due to these variables was small (1.2%).

**Table 5:** Multiple linear regression analysis of the PDRQ-9 German regressed on socioeconomic variables.

Criterion	Predictors	B	SE	Beta standardized	p	R^2^	Adjusted R^2^
PDRQ-9 total score	Age	.003	.001	.086	<.001	.014	.012
	Gender	.044	.030	.032	.146		
	Education	.024	.022	−.025	.284		
	Household income	.052	.013	.093	<.001		

Note: B  =  unstandardized regression coefficient; SE  =  standard error.

## Discussion

Summary of the main findings: We examined the internal and external validity of the PDRQ-9 German in a representative cross-sectional German population survey. We focused on participants who reported that they consulted with a PCP. The internal and external validity of the PDRQ-9 German were good.

Acceptance: The acceptance of the PDRQ-9 German was good, as only a few items were missing in the total sample. The acceptance rate of 99% is similar to those that were found with similar questionnaires in previous population surveys (e.g., 99.3%) [19].

Data quality: Similar to the Dutch [8] and Spanish studies [10], [11], ceiling effects were detected in the German PDRQ-9. The ability of the PDRQ-9 to discriminate within the upper region of satisfaction with PDR is insufficient [8], [11]. However, ceiling effects are inherent in all instruments that measure satisfaction with PDR [7]. Nevertheless, this problem should be noted. Furthermore, the results must be interpreted with caution, as the results of the CFA, multigroup analyses and correlation coefficients may be biased by the low variability in the PDRQ-9 scores found in the present study. When evaluating a questionnaire on the patient's perception of the helping attitude of his/her PCP, one should be aware that patients may provide a socially acceptable answer [8]. We attempted to eliminate this problem by assuring patients' anonymity and incorporating the PDRQ-9 into a survey without a specific focus. However, patients for whom a less positive doctor-patient relationship was expected (potential depressive disorder) showed significantly less satisfaction. This suggests that the PDRQ-9 might be able to discriminate between good and moderate doctor-patient relationships [8].

Reliability: The internal consistency of the PDRQ-9 German was high (α = .95), as it was in the Dutch (α = .94) [8], Spanish (α = .92 and .95) [10], [11] and Turkish (α = .91) [12] validation studies. Further, the psychometric properties of the German PDRQ-9 were very good with regard to the average variance extracted. From a statistical perspective, the corrected item-total correlations were very high (≥. 94). This raises the question of the usefulness of 9 different items and whether 1 item might be sufficient to measure the patient-doctor relationship. Conversely, the use of more than 1 item to measure a latent construct helps even out the measurement error of every single item. Additionally, the items address several related but distinct topics (for example, a trustful atmosphere, the helping attitude of the physician, and the time provided for consultations). Given that these are important aspects of the patient-doctor relationship, separate assessments are warranted.

Factorial structure: The current confirmatory analysis confirmed the factorial structure that van der Feltz-Cornelis et al. [8] and Mingote et al. [11] found using exploratory factor analysis. The PDRQ-9 German was shown to be unidimensional. The model fit indices showed that the assumption of a unidimensional scale fit the empirical data very well, with 1 exception. The CMIN/DF value indicated a relevant deviation between the empirical data and the model. This measure is sensitive to sample size. Thus, in the case of large sample sizes, even a small misspecification of the model can lead to its rejection. Therefore, we based our conclusion on the fit indices that are independent of the sample size, as described above (GFI, NFI, CFI, TLI, SRMR, and RMSEA). Additionally, the multigroup CFA revealed the strict factorial invariance of the model across men and women and for different age groups. Thus, the factor and observed mean scores as well as observed variances and covariances of these sub-groups can be compared in a statistical manner [26].

Construct validity: We confirmed our hypotheses concerning the convergent and the discriminative concurrent criterion validity. There was a moderate correlation between the mean PDRQ-9 satisfaction index and the satisfaction with pain treatment in persons with chronic pain, indicating convergent validity in a subsample of participants with chronic pain. The Turkish study found a moderate correlation of the PDRQ-9 Turkish total score with a generic instrument of patient satisfaction [12]. In testing the ability of the PDRQ-9 to discern difference, the Dutch study revealed higher total scores in primary care patients compared to patients from an Epilepsy clinic [8]. The current finding of minor satisfaction with PDR in depressed compared to non-depressed persons is in line with the results of the Heart and Soul study. Specifically, in outpatients with chronic coronary heart disease, depressive symptoms were associated with perceived deficits in doctor-patient communication, whereas medical comorbidities and disease severity were not associated with such deficits [17].

Associations of the PDRQ-9 and socioeconomic variables: The PDRQ-9 total scores slightly increased with rising age and household income. We found no gender differences. Similar to the present study, the validation study of the Spanish version did not find gender differences and detected a higher mean satisfaction index of elder people (aged>65 years) [10]. We speculate that seniors' greater satisfaction with PDR might depend on a more traditional role concept and/or a greater need for PCP consultation due to increasing morbidity. Additionally, we assume that participants with a higher income are more likely to be insured by private health insurance companies and, thus, may receive more attention (time, examinations) from their PCP. However, the impacts of age and income on the satisfaction index were very small.

Limitations: Although the response rate (57.5%) was comparable to those of other German health surveys [19], 42.5% of the persons who were addressed were non-responders. We do not have data to determine whether there were relevant differences between the participants of the survey and those who refused to participate. The data protection laws in Germany do not allow the assessment of the demographic data of non-responders. Additionally, our conclusions in regard to the convergent validity of the PDRQ-9 are based on a special subsample (people with chronic pain). Further empirical evidence is needed to support this assumption and to generalize the results of the present study. Another limitation is the lack of an assessment of discriminant validity, which was not addressed in the present study. Furthermore, we did not control the PDRQ-9 German using a social desirability questionnaire. Therefore, it remains possible that patients were biased toward a positive judgment in the assessment of their PCP.

Conclusions: Despite the limitation of the low variability in the PDRQ-9 scores, the German version of the PDRQ-9 is a brief and useful measure of the doctor-patient relationship from the patient's perspective. It has good psychometric properties and can be used for research in primary care, public health research and population surveys.

## Supporting Information

Appendix S1 PDRQ-9 German
(DOCX)

Table S1 Item score frequency
(DOCX)

Table S2 Frequency distribution of the PDRQ-9 total scores (percentile rank scores)
(DOCX)

## References

[1] Martin DJ, Garske JP, Davis MK (2000) Relation of the therapeutic alliance with outcome and other variables: a meta-analytic review. J Consult Clin Psychol 68: 438–50. 10883561

[2] Fuertes JN, Mislowack A, Bennett J, Paul L, Gilbert TC, et al (2007) The physician-patient working alliance. Patient Educ Couns 66: 29–36. 17188453 10.1016/j.pec.2006.09.013

[3] Thompson L, McCabe R (2012) The effect of clinician-patient alliance and communication on treatment adherence in mental health care: a systematic review. BMC Psychiatry 12: 87. 22828119 10.1186/1471-244X-12-87 PMC3528426

[4] Farin E, Gramm L, Schmidt E (2013) The patient-physician relationship in patients with chronic low back pain as a predictor of outcomes after rehabilitation. J Behav Med 2013 36: 246–58. 10.1007/s10865-012-9419-z 22476813

[5] Bensing JM (1991) Doctor-patient communication and the quality of care. An observation study into affective and instrumental behaviour in general practice. Academic dissertation. Utrecht: NIVEL.

[6] Ridd M, Shaw A, Lewis G, Salisbury C (2009) The patient-doctor relationship: a synthesis of the qualitative literature on patients' perspectives. Br J Gen Pract 59: e116–133. 19341547 10.3399/bjgp09X420248 PMC2662123

[7] Eveleigh RM, Muskens E, van Ravesteijn H, van Dijk I, van Rijswijk E, et al (2012) An overview of 19 instruments assessing the doctor-patient relationship: different models or concepts are used. J Clin Epidemiol 65: 10–15. 22118265 10.1016/j.jclinepi.2011.05.011

[8] van der Feltz-Cornelis CM, Van OP, Van Marwijk HW, De BE, Van DR (2004) A patient-doctor relationship questionnaire (PDRQ-9) in primary care: development and psychometric evaluation. Gen Hosp Psychiatry 26: 115–120. 15038928 10.1016/j.genhosppsych.2003.08.010

[9] Horvath A, Gaston L, Luborsky L (1993) The therapeutic Alliance and its measures. In: Miller NE, Barber JP, Docherty JP. Psychodynamic Treatment Research: A Handbook for Clinical Practice. New York: Basic Books. 247–273.

[10] Martín-Fernández J, del Cura-González MI, Gómez-Gascón T, Fernández-López E, Pajares-Carabajal G, et al (2010) [Patient satisfaction with the patient-doctor relationship measured using the questionnaire (PDRQ-9)]. Aten Primaria 42: 196–203. 20116893 10.1016/j.aprim.2009.09.026 PMC7024422

[11] Mingote Adán J, Moreno Jiménez B, Rodríguez Carvajal R, Gálvez Herrer M, Ruiz López P (2009) Psychometric validation of the Spanish version of the Patient-Doctor Relationship Questionnaire (PDRQ). Actas Esp Psiquiatr 37: 94–100. 19401857

[12] Mergen H, van der Feltz-Cornelis CM, Karoglu N, Mergen BE, Ongel K (2012) Validity of the Turkish patient-doctor relationship questionnaire (PDRQ-Turkish) in comparison with the Europe instrument in a family medicine center. HealthMed 6: 1763–1770.

[13] Hambleton RK (2005) Issues, designs and technical guidelines for adapting test into multiple languages and cultures. In: Hambleton RK, Merenda PF, Spielberger CD. Adapting Educational and Psychological Tests for Cross-Cultural Assessment. Mahwah, NJ: Erlbaum. 3–38.

[14] McHorney CA, Tarlov AR (1995) Individual-patient monitoring in clinical practice: are available health status surveys adequate? Qual Life Res 4: 293–307. 7550178 10.1007/BF01593882

[15] Fletcher A, Gore S, Jones D, Fitzpatrick R, Spiegelhalter D, et al (1992) Quality of life measures in health care. II: Design, analysis, and interpretation. BMJ 305: 1145–1148. 1463954 10.1136/bmj.305.6862.1145 PMC1883654

[16] International Association for the Study of Pain. Classification of chronic pain (1986) Pain suppl 3: S1–S226. 3461421

[17] Schenker Y, Stewart A, Na B, Whooley MA (2009) Depressive symptoms and perceived doctor-patient communication in the Heart and Soul study. J Gen Intern Med 24: 550–556. 19274477 10.1007/s11606-009-0937-5 PMC2669866

[18] Beck AT, Rush AJ, Shaw BF, Emery G (1979) Cognitive Therapy of Depression. New York: The Guilford Press.

[19] Häuser W, Glaesmer H, Schmutzer G, Brähler E (2012) Widespread pain in older Germans is associated with posttraumatic stress disorder and lifetime employment status—results of a cross-sectional survey with a representative population sample. Pain 153: 2466–2472. 23084003 10.1016/j.pain.2012.09.006

[20] Kroenke K, Spitzer RL, Jannett BW, Williams DSW, Löwe B (2009) An Ultra-Brief Screening Scale for Anxiety and Depression: The PHQ-4. Psychosomatics 50: 613–621. 19996233 10.1176/appi.psy.50.6.613

[21] Löwe B, Wahl I, Rose M, Spitzer C, Glaesmer H, et al (2011) A 4-item measure of depression and anxiety: validation and standardization of the Patient Health Questionnaire-4 (PHQ-4) in the general population. J Affect Disord 122: 86–95. 10.1016/j.jad.2009.06.019 19616305

[22] Fornell C, Larcker D (1981) Evaluating Structural Equation Models with Unobservable Variables and Measurement Error. J Marketing Res 18: 39–50.

[23] Arbuckle JL (2009) AMOS TM 18 User's Guide. Chicago: SPSS Inc.

[24] Schermelleh-Engel K, Moosbrugger H (2003) Müller (2003) Evaluating the fit of structural equation models: Tests of significance and descriptive goodness-of-fit measures. MPR Online 8: 23–74.

[25] Hu L, Bentler PM (1998) Fit indices in covariance structure modeling: Sensitivity to underparameterized model misspecification. Psychol Methods 3: 424–453.

[26] Gregorich SE (2006) Do self-report instruments allow meaningful comparisons across diverse population groups? Testing measurement invariance using the confirmatory factor analysis framework. Med Care 44: 78–94. 10.1097/01.mlr.0000245454.12228.8f PMC1808350 17060839

[27] Cheung GW, Rensvold RB (2002) Evaluating goodness-of-fit indexes for testing measurement invariance. Struct Equ Modeling 9: 233–255.

[28] Harlow LL (2005) The essence of multivariate thinking. Basic theories and methods. Harvard: Lawrence Erlbaum Assoc.

[29] Federal Statistical Office. Zensus 2011. https://www.destatis.de/DE/PresseService/Presse/Pressekonferenzen/2013/Zensus2011/Pressebroschuere_zensus2011.pdf?__blob=publicationFile; Accessed 2013 Sep 2.

