# Supplementary material for: Validation of the Patient-Doctor-Relationship Questionnaire (PDRQ-9) in a Representative Cross-Sectional German Population Survey
Source: PLoS One. 2014 Mar 17;9(3):e91964. doi: 10.1371/journal.pone.0091964 (PMC3956823; doi:10.1371/journal.pone.0091964)
Supplement: Appendix S1 — PDRQ-9 German (DOCX) [file pone.0091964.s001.docx]

**Appendix S1: PDRQ-9 German**

|  | **Fragebogen zur Arzt-Patient-Beziehung (PDRQ-9):**  Nachfolgend finden Sie neun Aussagen, die man über seinen Hausarzt machen kann. Bitte markieren Sie zu jeder Aussage eine Zahl, die auf Ihren Hausarzt zutrifft. | trifft überhaupt nicht zu | trifft  kaum  zu | trifft teilweise zu | trifft weit-gehend zu | trifft voll und ganz zu |
| --- | --- | --- | --- | --- | --- | --- |
| 1. | Ich glaube, dass mein Arzt mir hilft | ➀ | ➁ | ➂ | ➃ | ➄ |
| 2. | Mein Arzt hat genug Zeit für mich | ➀ | ➁ | ➂ | ➃ | ➄ |
| 3. | Ich vertraue meinem Arzt | ➀ | ➁ | ➂ | ➃ | ➄ |
| 4. | Ich habe das Gefühl, dass mein Arzt mich versteht | ➀ | ➁ | ➂ | ➃ | ➄ |
| 5. | Meinem Arzt ist sehr daran gelegen, mir zu helfen | ➀ | ➁ | ➂ | ➃ | ➄ |
| 6. | Mein Arzt und ich stimmen bezüglich der Ursachen meiner Beschwerden überein | ➀ | ➁ | ➂ | ➃ | ➄ |
| 7. | Ich kann mit meinem Arzt gut reden | ➀ | ➁ | ➂ | ➃ | ➄ |
| 8. | Ich bin mit der Behandlung durch meinen Arzt zufrieden | ➀ | ➁ | ➂ | ➃ | ➄ |
| 9. | Ich empfinde meinen Arzt als offen und zugänglich | ➀ | ➁ | ➂ | ➃ | ➄ |
