# Supplementary material for: Validation of the Patient-Doctor-Relationship Questionnaire (PDRQ-9) in a Representative Cross-Sectional German Population Survey
Source: PLoS One. 2014 Mar 17;9(3):e91964. doi: 10.1371/journal.pone.0091964 (PMC3956823; doi:10.1371/journal.pone.0091964)
Supplement: Table S1 — Item score frequency (DOCX) [file pone.0091964.s002.docx]

**Table S6: Item score frequency**

| Item | Frequency (%) | | | | |
| --- | --- | --- | --- | --- | --- |
|  | 1 | 2 | 3 | 4 | 5 |
| 1 My PCP helps me | 7 (0.3) | 67 (2.9) | 323 (14.2) | 1.019 (44.8) | 859 (37.8) |
| 2 My PCP has enough time for me | 15 (0.7) | 189 (8.3) | 571 (25.1) | 870 (38.2) | 630 (27.7) |
| 3 I trust my PCP | 9 (0.4) | 52 (2.3) | 319 (14.0) | 968 (42.5) | 923 (40.6) |
| 4 My PCP understands me | 18 (0.8) | 79 (3.5) | 350 (15.4) | 1.021 (44.9) | 804 (35.3) |
| 5 My PCP is dedicated to help me | 6 (0.3) | 40 (1.8) | 287 (12.6) | 964 (42.4) | 976 (42.9) |
| 6 My PCP and I agree about the nature of my medical symptoms | 11 (0.5) | 87 (3.8) | 436 (19.2) | 951 (41.8) | 777 (34.2) |
| 7 I can talk to my PCP | 9 (0.4) | 99 (4.4) | 368 (16.2) | 892 (39.2) | 907 (39.9) |
| 8 I feel content with my PCP’s treatment | 20 (0.9) | 76 (3.3) | 319 (14.0) | 987 (43.4) | 872 (38.3) |
| 9 I find my PCP easily accessible | 11 (0.5) | 75 (3.3 | 347 (15.3) | 929 (40.8) | 913 (40.1) |

Note: PCP = primary care physician; 1 = not at all appropriate; 2 = somewhat appropriate; 3 = appropriate; 4 = mostly appropriate; 5 = totally appropriate
