# Supplementary material for: Validation of the Patient-Doctor-Relationship Questionnaire (PDRQ-9) in a Representative Cross-Sectional German Population Survey
Source: PLoS One. 2014 Mar 17;9(3):e91964. doi: 10.1371/journal.pone.0091964 (PMC3956823; doi:10.1371/journal.pone.0091964)
Supplement: Table S2 — Frequency distribution of the PDRQ-9 total scores (percentile rank scores) (DOCX) [file pone.0091964.s003.docx]

**Table S7: Frequency distribution of the PDRQ-9 total scores (percentile rank scores)**

| **Raw score** | Percent rank | **Raw score** | Percent rank | **Raw score** | Percent rank |
| --- | --- | --- | --- | --- | --- |
| **9** | 0.0 | **22** | 2.1 | **35** | 34.5 |
| **10** | 0.1 | **23** | 2.9 | **36** | 45.2 |
| **11** | 0.1 | **24** | 4.3 | **37** | 50.1 |
| **12** | 0.1 | **25** | 5.1 | **38** | 56.3 |
| **13** | 0.2 | **26** | 6.2 | **39** | 61.7 |
| **14** | 0.47 | **27** | 9.2 | **40** | 66.8 |
| **15** | 0.4 | **28** | 10.8 | **41** | 71.5 |
| **16** | 0.5 | **29** | 12.5 | **42** | 75.5 |
| **17** | 0.5 | **30** | 14.9 | **43** | 79.6 |
| **18** | 0.8 | **31** | 17.3 | **44** | 83.7 |
| **19** | 0.9 | **32** | 20.1 | **45** | 100.0 |
| **20** | 1.2 | **33** | 24.0 |  |  |
| **21** | 1.8 | **34** | 28.8 |  |  |
